# Recycled-Oil-Based Polyurethane Modified with Organic Silicone for Controllable Release of Coated Fertilizer

**DOI:** 10.3390/polym11030454

**Published:** 2019-03-10

**Authors:** Qian Wang, Fuping Dong, Jun Dai, Qingpo Zhang, Meng Jiang, Yuzhu Xiong

**Affiliations:** Department of Polymer Materials and Engineering, College of Materials and Metallurgy, Guizhou University, Guiyang 550025, China; Wqian1127@163.com (Q.W.); fpdong@gzu.edu.cn (F.D.); dj03012@163.com (J.D.); zqp2012@foxmail.com (Q.Z.); jiangmeng1994@126.com (M.J.)

**Keywords:** recycled oil, polyurethane, coating materials, three-dimensional networks, sustained release

## Abstract

Fertilizer is very important for increasing food yield, but the extensive use of fertilizer will cause environmental pollution. To enhance the effectiveness of fertilizer, we developed the double organic silicone-modified recycled-oil-based polyurethane as a coating material to prepare degradable polymer coating urea for constant fertilizer release. The moisture, heat resistance, and sustained release properties of polyurethane coating materials were investigated by modification with hydroxyl-terminated polydimethyl silicone (HTPMS) and γ-Aminopropyl triethoxy silane (KH550). The content and distribution of the siloxane groups were effectively controlled by adjusting the content ratio of two kinds of organosilicon. Meanwhile, the organic–inorganic hybrid structure was further controlled to form three-dimensional networks with a uniform distribution and a small scale. The moisture and heat resistance of polyurethane were thus improved, resulting in reduced porosity and an excellent sustained release performance. Observably, the best sustained release property of modified polyurethane coated urea was obtained when the ratio of KH550 to HTPMS is 0.3:0.7.

## 1. Introduction 

The requirement for productive and sustainable agriculture is highly urgent with the rapid growth of the world population [1]. Fertilizer is one of the important input materials for crop production [2]. However, the nutrient utilization efficiency from pure fertilizers is markedly reduced due to the volatilization and leaching of nutrients [3]. According to statistics [4,5], the effective utilization of nitrogen (N) fertilizer is very low (30–50%). The wasting of N fertilizer not only causes economic losses but also negatively affects our environment [6,7]. Therefore, great efforts have been made to develop polymer coated fertilizers (PCF) with controlled-release behaviors. Notably, PCF can not only enhance crop yield but also reduce nutrient loss to the surrounding environment [8]. Previous research work [9] indicated that the application of PCF could increase the average utilization rate of fertilizer by 60%–80%. Especially, the nutrient components of fertilizer lost by leaching and infiltration can be reduced, and this can effectively reduce the frequency of fertilizer use compared with commercial fertilizers. In addition, the water quality will also be will improved because of the controlled-release behavior of PCF [10].

The use of controlled-release fertilizer (CRF), especially PCF, play an important role in the nutrient release profiles by providing obstacles to the mixing of nutrients with water [11]. The conventionally fertilizer-coating polymers, such as organic solvent-based polymers, thermosetting polymers formed from two or more components, and polymer latex in which the continuous phase is water [12], are high cost and environmentally harmful. They show a decreased nutrient content in the coated fertilizer. Recently, biodegradable materials, which are environmentally friendly, have shown good results in maintaining the optimal controlled-release rate [13]. Particularly, coated fertilizer with biodegradable materials can not only enhance the absorbed nutrients in the fertilizer but also avoid the decrease of physical and chemical properties of the soil caused by nutrient loss [14,15,16,17]. At the same time, the soil quality can be maintained efficiently because of the degradability of the coating material.

Hogwash oil has a wide range of sources and a low cost. The large output is very harmful to people’s lives. For instance, more and more attention has been paid to the resource utilization of hogwash oil. Typically, hogwash oil is widely used in the field of surfactant, cleaning agent, biodiesel, and so on [18,19,20]. In other words, the hogwash oil can also be defined as the recovered oil; however, the recovered oil is often casually treated and ignored because it is innocuous and harmless, which causes waste of some resources and pollutes the environment. As our continued research is interested in the resource utilization of hogwash oil [21,22,23], herein, we developed the double organic silicone-modified recycled-oil-based polyurethane as a coating material to prepare degradable polymer coating urea for constant fertilizer release. The moisture, heat resistance, and sustained release properties of polyurethane materials (PUs) were investigated by modifying with hydroxyl-terminated polydimethyl silicone (HTPMS) and γ-Aminopropyl triethoxy silane (KH550). The results indicated that HTPMS improves the hydrophobicity of PUs for its low cohesive energy density and good water resistance [24] and that KH550 improves the mechanical, electrical, water resistance, and aging resistance of the polymer [25]. Moreover, the presence of silicon plays an important role in all functions of the body and is directly related to the absorption of minerals. This degradable polymer coating urea is promising in protecting agricultural/ecological environment and also could make full use of recovered oil.

## 2. Experimental 

### 2.1. Materials

The recovered oil was purchased from Bijie City Restaurant in Guizhou Province. Phosphoric acid (AR, ≥85.0%) was obtained from Jiayu fine chemical Co., Ltd. (Tianjin, China). Sodium hydroxide (AR, ≥96.0%) was provided by Yongda Chemical Reagent Co., Ltd. (Tianjin, China). Sodium chloride (AR, ≥99.5%) was purchased from Cormico Chemical Reagent Co., Ltd. (Tianjin, China). Acticarbon was obtained from Kwangfu Technology Development Co., Ltd. (Tianjin, China). Diphenyl methane diisocyanate (MDI-50) was bought from Shandong Jiaying Chemical Technology Co., Ltd. (Shandong, China). Hydroxyl-terminated poly dimethyl silicone (HTPMS) was provided by Hubei Xinsihai chemical Co., Ltd. (Hubei, China). γ-Aminopropyl triethoxy silane (KH550) was obtained from Green Wei Plastic Products Co., Ltd. (Dongguan, China). Peroxyacetic acid was purchased from Dongguan Xilong science co., Ltd. (Guangdong, China). Ethylene glycol (AR) was provided by Chengdu Jinshan chemical reagent co., Ltd. (Chengdu, China). Aluminium chloride (AR, ≥99%) was purchased from Aladdin. Urea (ca. 2 mm in diameter and with 46.4% N) was obtained from Jiangsu Jinmei Hengsheng Chemical Co., Ltd. (Jiangsu, China).

### 2.2. Purification of Recovered Oil

The major components of oils include high-grade fatty acid glycerides, various unsaturated acids, etc. Therefore, this work mainly utilized the recovered oils as raw material, and their degradation products formed during regular use, including food impurities, gels, etc., were removed by filtration to remain as the main components of pure oil. A certain amount of recovered oil was filtered several times and treated with phosphoric acid (0.8 wt.% of oil) for 8 h at 70 °C with stirring in an oil bath. Then, distilled water (10 wt.% of oil) was added to the oil, and the upper oil part was obtained after centrifugation. The oil was heated at 100 °C for a period of time and was neutralized with NaOH solution. A NaCl aqueous solution (6 wt.% of Oil) was added to the oil and stirred for 6 h, and then distilled water (10 wt.% oil) was added under stirring. After centrifugation, the obtained upper oil sample was obtained and heated at 110 °C for 1 h to remove water, and then an appropriate amount of acticarbons were added to remove the impurities. Finally, the recovered oil (RO) was obtained by hot filtration with an oil temperature at about 70 °C.

### 2.3. Premodification of Recycled Oil

The recovered oil was premodified according to the procedures reported by the literature [26,27,28] with slightly modification. Typically, 150 g of recovered oil was added to a 3L-four-necked reaction kettle equipped with a mechanical stirrer, thermometer, and water condenser. The system was heated at 80 °C in an oil bath under a nitrogen environment. Then, 25 g of peroxyacetic acid and 1 g diluted sulfuric acid (3 wt.%) were added to the system. After stirring for 2 h, the diluted NaOH aqueous solution was added into the system until the oil became neutral. After washing several times with saturated NaCl solution and distilled water, the epoxidation recycled oil (ERO) was obtained by vacuum distillation. Then, 75 mL ethylene glycol and 7.5 g aluminum chloride were added to ERO and heated for 2 h at 95 °C under nitrogen protection. Finally, the system was washed with distilled water to remove the unreacted ethylene glycol, and alcoholization-recycled oil (ARO) was obtained after vacuum distillation.

### 2.4. Preparation of KH550/HTPMS Modified PCUs

**Figure 1:**
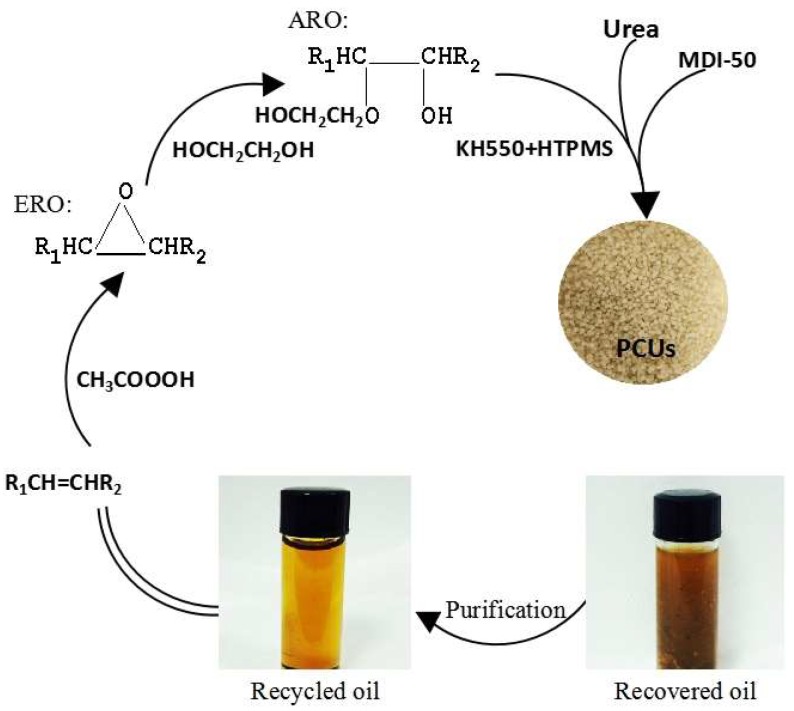
The preparation process of the KH550/HTPMS modified polyurethane materials coated urea (PCUs).

KH550/HTPMS (20 wt.% of oil) was added into the ARO with stirring at 80 °C for 2 h. The weight ratios of the KH550 and HTPMS were adjusted from 0.1:0.9, 0.2:0.8, 0.3:0.7, 0.4:0.6, to 0.5:0.5, respectively. The coating process was performed in a BYC-300 coating machine with the rotation speed and temperature of the coating drum at 60 rpm and 60 °C, respectively. As illustrated in Figure 1, 150 g urea was added to the BYC-300 coating machine, followed by the addition of 5 g of the mixture of KH550/HTPMS/ARO, and then 2.7 g MDI-50 was added into the coating machine. The polyurethane materials coated urea (PCUs) was obtained after reaction for 10 min and then kept for another 24 h in a vacuum oven at 80 °C. For the control experiment, the double organic silicone-modified polyurethane materials (K-H-PUs) were obtained with the same conditions without urea. Especially, no final purification step was performed here because the isocyanate groups are excessive in comparison with the other reactive groups during the preparation process, and the ratio of excess isocyanate groups in the system is constant for each experiment, which ensures the reproducibility of the experiment and has little effect on the materials. The properties of the final materials depend on the synthesized polyurethane.

### 2.5. Nitrogen Release from PCUs

The nitrogen release experiments were performed according to the reference reported by Oertli et al. [29] with slight modification. Typically, three replicates of 5 g of the PCUs sample and 200 g of soil were mixed uniformly, and then, the mixtures were put into a polyvinyl chloride pipe with a diameter of 5 cm and a length of 60 cm. At the bottom of PVC tube, a layer of thin gauze was equipped for filtration. At certain time intervals (every 5 days until 30 days), 100 mL of distilled water was added into the tube and the filtrate through the gauze was collected. In order to investigate the release behavior of the fertilizer, the nitrogen amount released from the PCUs samples was determined using UV-Vis spectroscopy (TU-1810) by measuring the absorbance at the wavelength of λ = 440 nm [30]. The amount of the released nitrogen was calculated according to the absorbance of the solution.

### 2.6. Characterization 

#### 2.6.1. Fourier Transform Infrared Spectroscopy (FTIR) Analysis

A Fourier transform infrared spectroscopy of KBr powder-pressed pellets was recorded on a Nicolet 6700 FTIR spectrophotometer (NEXUS6700, Thermo Nicolet Co. Ltd., Beijing, China) in the frequency range from 4000 to 400 cm^−1^ with the spectral resolution of 1 cm^−1^.

#### 2.6.2. Liquid Nuclear Magnetic Resonance (NMR) Spectrometer Analysis

The surface structures of the PUs and K-H-PUs coatings were analyzed with an Avance Bruker 400 liquid nuclear magnetic resonance spectrometer (Ascend400, Bruker Technology Co., Ltd., Beijing, China). The solvent in the experiment was *N*,*N*-Dimethylformamide-*d*_7_.

#### 2.6.3. X-ray Photoelectron Spectroscopy (XPS) Analysis

The surface of the PUs was analyzed using a Thermo K-Alpha Multifunctional imaging electron spectrometer (K-Alpha, Thermo Scientific Co. Ltd., Waltham, MA, USA), incorporating a hemispherical electron energy analyzer. The incident radiation was monochromatic Al Ka X-ray. The peak fit of the data was then performed using XPS Peak software.

#### 2.6.4. Water Contact Angles (WCAs) Analysis

Five replicates of water contact angle measurements were performed on a Kruss DSA25 machine (Hamburg, Germany) (using 3 μL of water droplets).

#### 2.6.5. Scanning Electron Microscopy (SEM) Analysis

The sample morphologies were observed by scanning electron microscopy (SEM, JSM-7500F, Amberlai Scientific Instruments Co., Ltd., Shanghai, China).

#### 2.6.6. Determination of Water Permeability

The coating water permeability was determined based on the weight difference between the wet sample (*W*_w_) and dry sample (*W*_d_) as follows [31]:

$$\varepsilon =\frac{(W_{w}-W_{d})/\rho_{w}}{(W_{w}-W_{d})/\rho_{w}+W_{d}/\rho_{d}}\times 100\%$$

where *ρ*_w_ and *ρ*_d_ are the densities of water and polymer, respectively. The coating water permeability was determined based on the average of five parallel experiment.

#### 2.6.7. Thermogravimetric Analysis (TGA)

A TGA analysis was carried out using a thermogravimetric analyzer (STA449F3, Germany Netzsch Instrument Manufacturing Co., Ltd., Selb, Germany) under nitrogen atmosphere with a flow rate of 50 mL/min from room temperature to 700 °C with a heating rate of 10 °C/min and weighing analysis accuracy degree is 0.1 μg.

## 3. Results and Discussion

### 3.1. Structural Analysis of the PUs and the K-H-PUs

**Figure 2:**
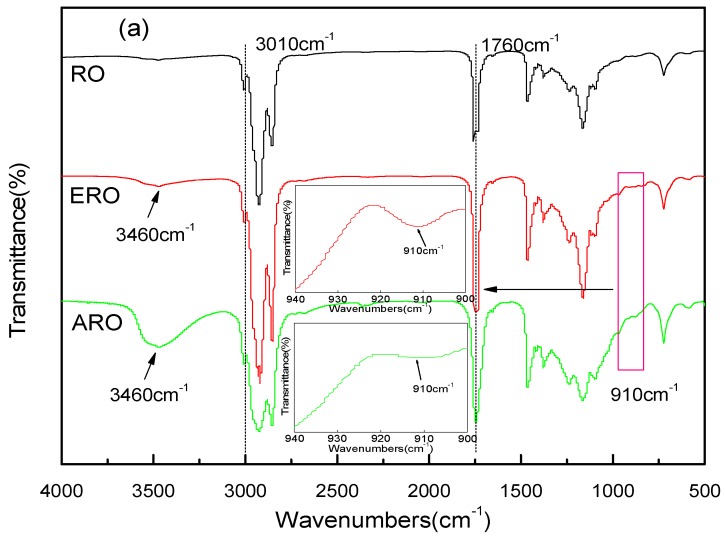

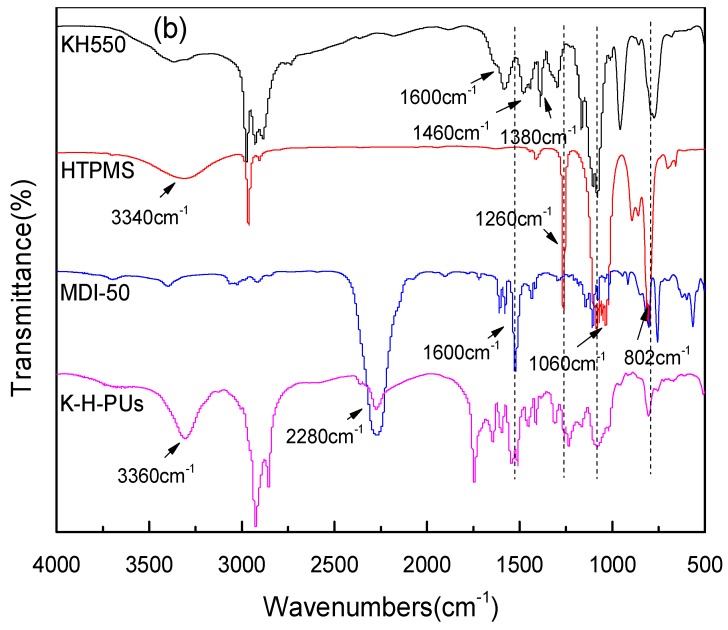
(**a**) The FTIR spectra of recycled oil (RO), epoxidation recycled oil (ERO), and alcoholization recycled oil (ARO) and (**b**) the KH550/HTPMS modified PUs.

The FTIR spectra of RO, ERO, and ARO are shown in Figure 2a. In the FTIR spectrum of RO, the characteristic peaks at 3010 cm^−1^ and 1760 cm^−1^ were attributed to the C=C–H and C=O stretching vibrations, respectively. In the spectrum of ERO, the new peaks at 910 cm^−1^ (Figure 2a ERO at high resolution) and 3460 cm^−1^ were assigned to the stretching vibration of epoxy groups and hydroxyl group [26], respectively. At the same time, the peak at 3010 cm^−1^ was weakened, demonstrating that C=C–H bonds were partially oxidized to form epoxy groups. In the spectrum of ARO, the characteristic peak at 910 cm^−1^ disappeared and the peak at 3460 cm^−1^ was obviously enhanced, indicating that a small amount of hydroxyl groups transformed from epoxy group by a ring-opening reaction, which means that the RO was successfully modified. 

In the FTIR spectrum of KH550 (Figure 2b), the characteristic peaks at 1380 cm^−1^, 1460 cm^−1^ and 1600 cm^−1^ were attributed to the stretching or bending vibration of C–N, C–H, and N–H, respectively [32]. In the FTIR spectrum of HTPMS, the peaks at 802 cm^−1^, 1060 cm^−1^, 1260 cm^−1^, and 3340 cm^−1^ were assigned to the Si–CH_3_ rocking vibration, Si–O–Si and Si–CH_3_ symmetry bending, and –OH stretching vibration [31]. In the FTIR spectrum of MDI-50, the peaks at 2280 cm^−1^ and 1600 cm^−1^ indicate the –NCO and benzene ring skeleton. In the FTIR spectrum of K-H-PUs, the peak at 2280 cm^−1^ assigned for the –NCO group on MDI-50 was greatly weakened, and a new peak at the 3360 cm^−1^ assigned for the –NH group appeared, which means the formation of PUs. Furthermore, the characteristic peaks at 802 cm^−1^, 1060 cm^−1^, and 1260 cm^−1^ mean that the organic silicone was successfully modified on PUs.

**Figure 3:**
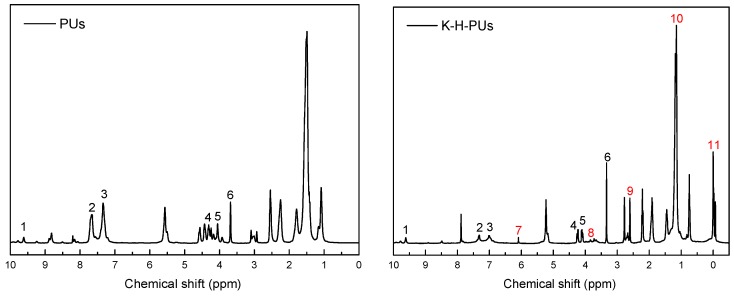
The ^1^H NMR spectra of coating polyurethane materials (Pus) and double organic silicone-modified polyurethane materials (K-H-PUs).

**Scheme 1:**
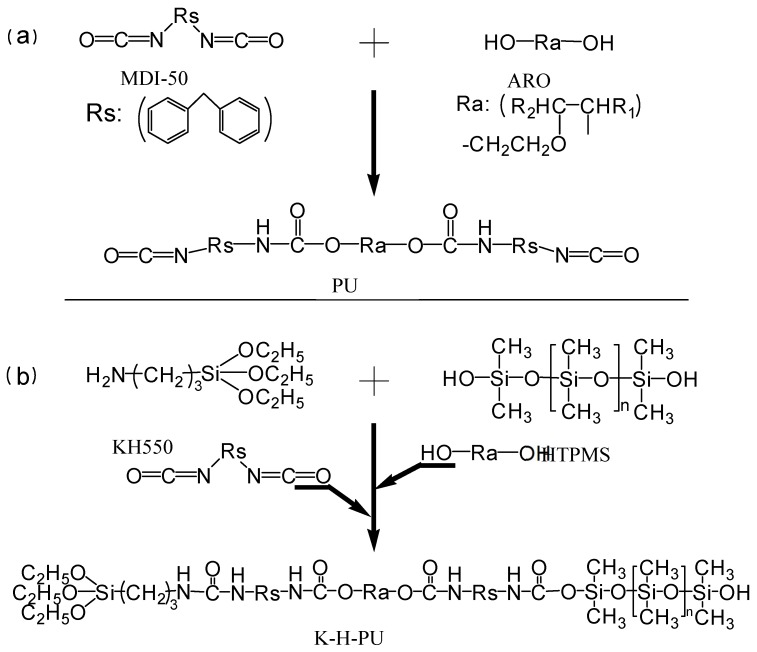
The synthesis route of before (**a**) and after (**b**) the modification of PUs.

To further confirm the formation of coating PUs, the ^1^H NMR was employed (Figure 3). The peaks near δ = 7.5 ppm (2 and 3) indicate two kinds of hydrogen absorption peaks over benzene ring of isocyanate, respectively [33]. The peaks at δ = 4.1 ppm (5) represent the hydrogen of methylene carbons connected with benzene rings. In addition, the protons signals of 4 and 6 are from the methylene at different reaction ends of modified recycled oil. These results further support the successful fabrication of PU coatings. Notably, the peak of 7 appears in the ^1^H NMR spectrum of modified PUs at δ = 6.1 ppm, indicating that the hydrogen absorption peak of the amide bond produced by the reaction of the amino group of KH550 with the isocyanate group. Moreover, the proton characteristic signals of KH550 and HTPMS could also be clearly found in the ^1^H NMR of K-H-Pus; it shows that the two coatings modified with organic silicone were successfully prepared through the complete polymerization reaction as shown in Scheme 1.

### 3.2. Surface Elemental Composition Analyses

**Figure 4:**
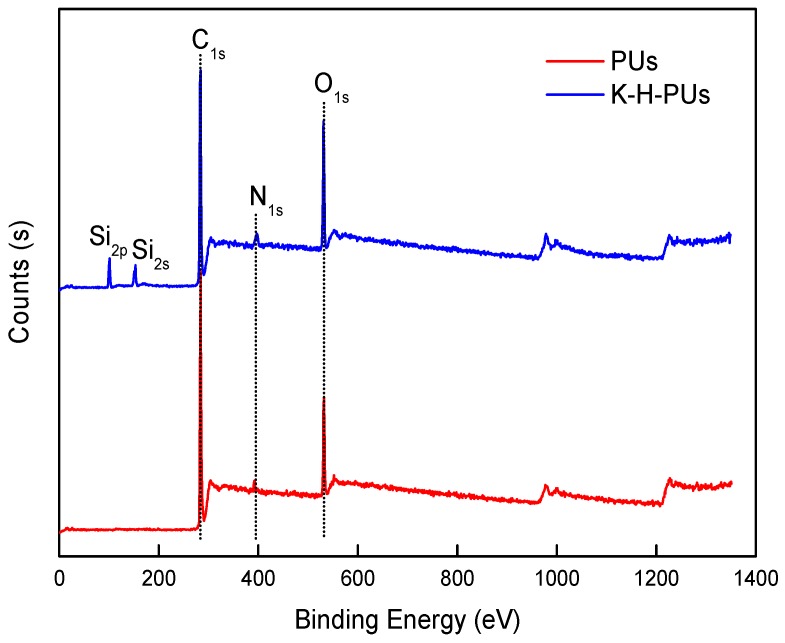
The survey XPS of before and after the modification of PUs.

Figure 4 shows the XPS survey of PUs before and after the modification by KH550/HTPMS. Both PUs and K-H-PUs show three identical peaks at 284, 399, and 532 eV, which were distributed to carbon (1s), nitrogen (1s), and oxygen (1s), respectively. In contrast, two peaks at 101 and 153 eV were assigned to silicon (2p) and silicon (2s) in K-H-PUs [34]. Furthermore, we found that the atomic concentration ratio of O/C, N/C, and Si/C increased from 17.78% to 24.30%, 3.34% to 5.64%, and 0 to 13.66%, respectively (Table 1). The result of the XPS indicated that PUs are successfully modified by organic silicone, which is consistent with the FTIR and ^1^H NMR.

**Figure 5:**
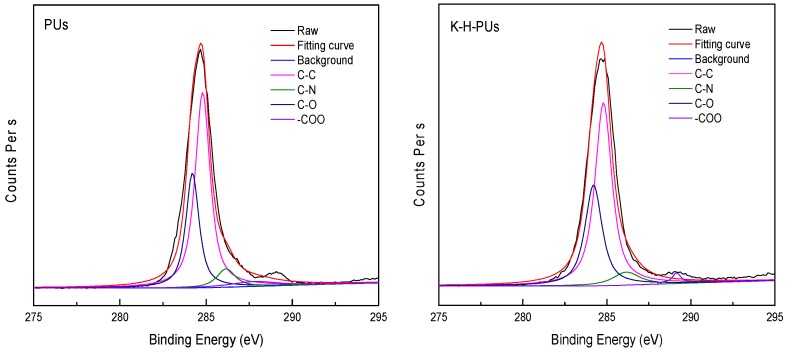
The XPS C1s peak of PUs before and after modification by KH550/HTPMS.

As shown in Figure 5, the C1s peaks of the XPS spectra for PUs were fitted by a multipeak Lorentzian fitting program (XPS peak). The surface binding states of PUs before and after modification are C–C, C–N, C–O, and –COO. After it was modified by KH550/HTPMS, the C–O contents obviously dropped from 28.97% to 24.55%. Conversely, the C–N and –COO content increased from 14.80% to 16.05% and from 12.53% to 14.78%, respectively (Table 2). Based on these results, we confirmed that the –NCO groups of MDI-50 can react with the active groups of oil or double organic silicone.

### 3.3. Thermal Stability Analyses

**Figure 6:**
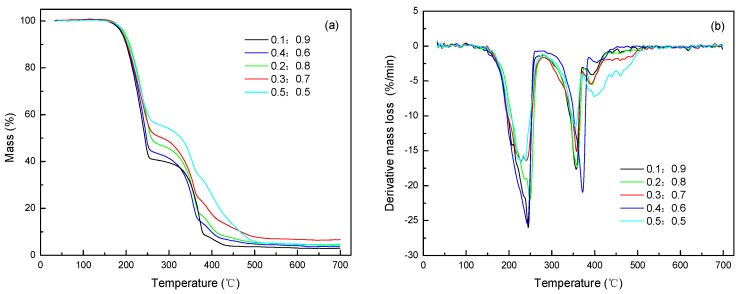
The TGA (**a**) and DTG (**b**) curves for PCU containing different ratios of KH550/HTPMS.

The stability properties and degradation behavior were also studied by techniques of TGA. As shown in Figure 6a. The results show two different degradation stages starting at about 170 °C and 320 °C, which mainly comes from the decomposition of PUs and urea [35]. In addition, the derivative thermogravimetry (DTG) curves illustrate this degradation process. As show in Figure 6b, the first degradation stage of PUs occurred at 250–300 °C, and isocyanate and alcohol were produced; the second degradation stage occurred at 420–510 °C, and secondary amine, olefin, and CO_2_ were produced [36]. As show in Table 3, the thermal stability properties of PU shells were apparently improved after modification with KH550/HTPMS. For example, the temperatures for 5 % and 50 % mass loss (according to the literature [37], for unmodified shells, they are 189.8 °C and 238.8 °C) were increased. The maximum degradation rate of PUs was at about 360 °C (Table 3). Particularly, the decomposition temperature of PCU reached the maximum when the ratio of KH550:HTMS was 0.5:0.5; it was possibly because the Si–O bond required more energy to dissociate than the C–C and C–O bond.

### 3.4. Water Contact Angle

**Figure 7:**
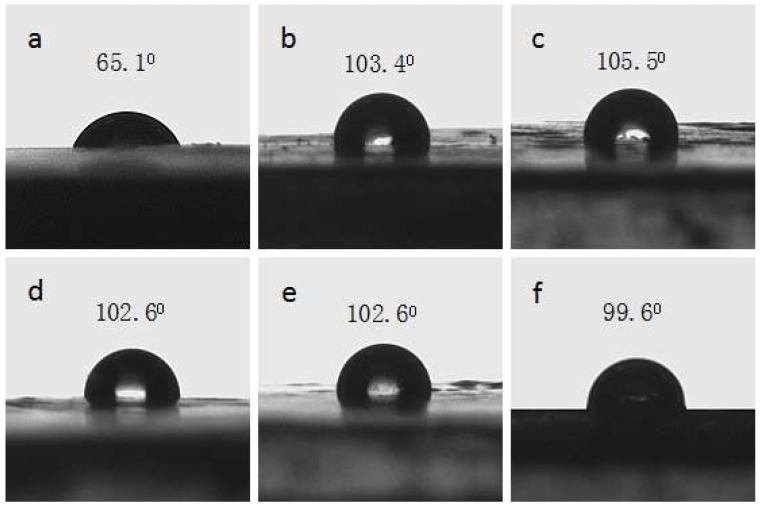
The water contact angle of PCU shells containing different ratios of KH550/HTPMS: (**a**) 0, (**b**) 0.1:0.9, (**c**) 0.2:0.8, (**d**) 0.3:0.7, (**e**) 0.4:0.6, and (**f**) 0.5:0.5.

The water contact angle images are shown in Figure 7. It was found that the modified coating shell illustrated improves hydrophobic property. Compared to the unmodified shells, which the average value was 65.1° for contact angle, PCU shells modified with KH550/HTPMS have much higher contact angles of 103.4°, 105.5°, 102.6°, 102.6°, and 99.6° with the ratios of KH550/HTPMS as 0.1:0.9, 0.2:0.8, 0.3:0.7, 0.4:0.6, and 0.5:0.5, respectively (Figure 7). The improved hydrophobicity is respected to be very important for the constant release behavior of the fertilizer. (The standard deviations are 0.84, 0.78, 0.64, 0.92, and 0.89, respectively.)

### 3.5. Macroscopic and Microscopic Morphology

**Figure 8:**
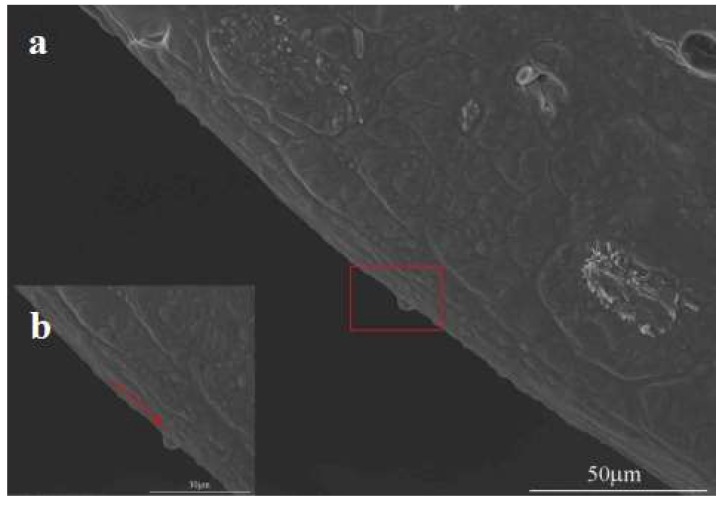
A SEM image of the section containing the interface between the urea and PUs. (**a**) 2000×, (**b**) 5000×.

**Figure 9:**
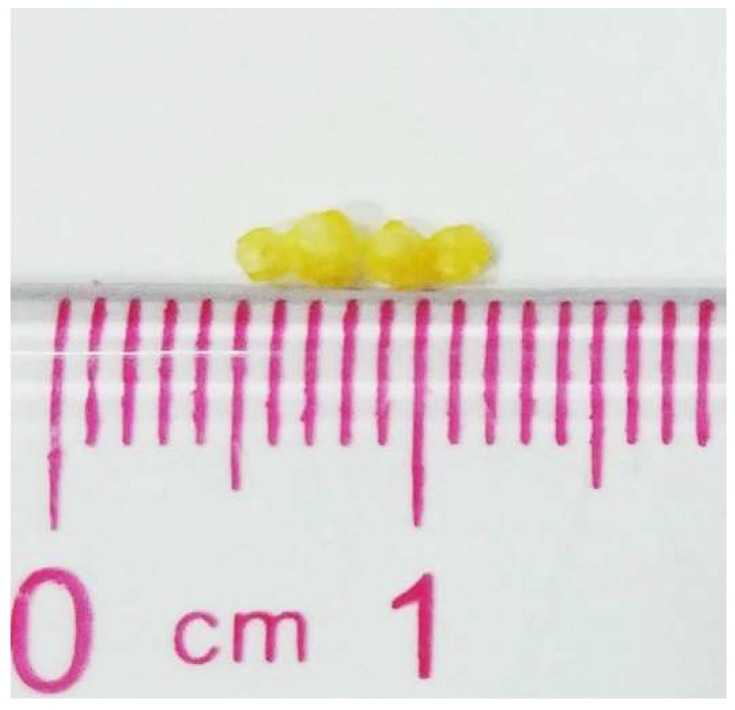
A photograph of the PCU coatings after the release of urea in an aqueous solution.

As shown in Figure 8, the cohesive film with a thickness of approximately 10 μm was observed. Meanwhile, the existence of orientation along the particle surface was observed (arrow in Figure 8b), originating from the sample preparation process, in which the sample received the force only in one direction during the coating process. Furthermore, the best interfacial could be seen clearly between the film and the urea granules. There was a protrusion on the surface of the coating which was caused by a brittle fracture process. The photograph of Figure 9 shows that the core urea particles fertilizers were released completely in aqueous solution and only left the PU coating materials. The integrity of the polyurethane coating remained intact after the complete release of urea. It is noted that the polyurethane has a good film-forming property and a complete coating on the surface of urea particles.

### 3.6. Water Permeability of the Polymer Coating Films

**Figure 10:**
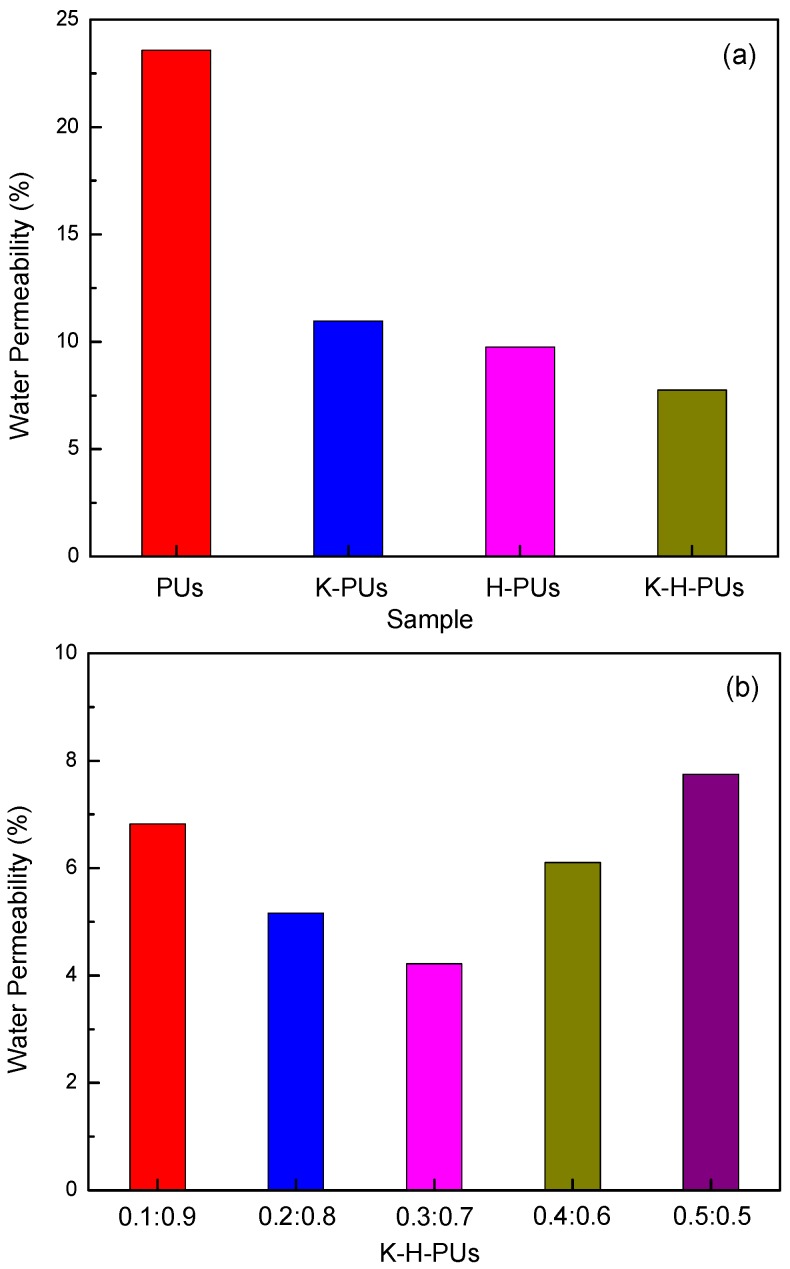
The effect of KH550/HTPMS modification on the coating water permeability: (**a**) different organic silicon and (**b**) different ratio of KH550/HTPMS.

The water permeabilities of coating materials before and after the modification of PUs are shown in Figure 10. The unmodified shells present a 23.6% water permeability. In contrast, the water permeabilities of PCU shells were significantly reduced to 11.0%, 10.0%, and 7.8%, after the modification by KH550, HTPMS, and KH550/HTPMS, respectively (Figure 10a). The water permeability varies with the ratio of KH550/HTPMS, and when the ratio of KH550/HTPMS is 0.3:0.7, the water permeability reaches the minimum (Figure 10b). The decreased water permeability was associated with the increased cross-linking degree of modified PUs and the more compaction of cross-linking network structure. In fact, the cross-linking degree was increased significantly after the modification of the organic silicone, especially after the modification of the double organic silicone. On the other hand, the lowest water permeability of PU modified by KH550/HTPMS may also come from the different molecular weight of modifiers that makes the densification of cross-linking network structure of the modified PUs. The inactive components, the coating that reacts with the curing agents to form the PUs film, were fixed stable in the coating shells [34]. This low water permeability of the modified PU was respected to be one of the reasons to enhance the slow-release behavior of the fertilizer.

### 3.7. Release Profiles of PCU

**Figure 11:**
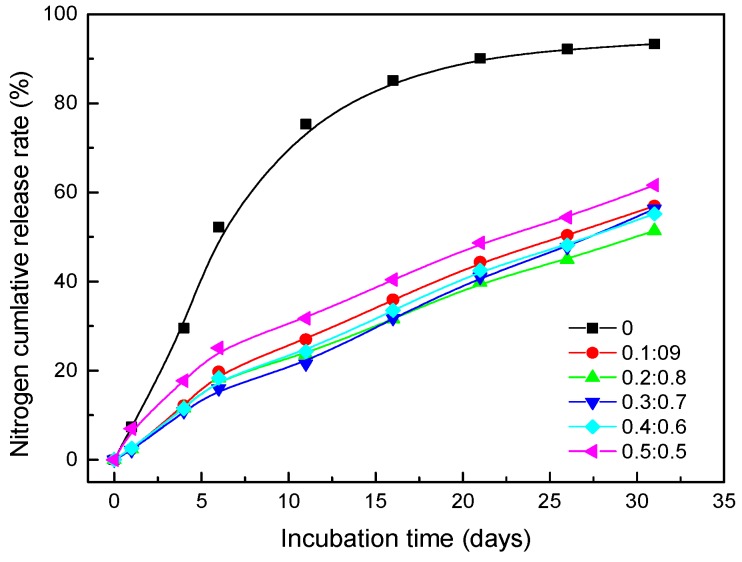
The cumulative nitrogen release curves of PCU (with different ratio of KH550/HTPMS).

The nutrient release profile, estimated as the nitrogen cumulative dissolution rate versus time, was essential for the fertilizer release behavior with the modified PU as coating films. Figure 11 illustrates the effect of coating films on the nutrient release profile with the unmodified PUs and the PUs modified with KH550/HTPMS. Compared with the unmodified PUs, PCU shells modified by KH550/HTPMS have a much slower nitrogen release rate. This mainly comes from the increased hydrophobicity, the increased thermal stability, and the reduced water permeability. Especially, a higher thermal stability shows stronger chemical bonds, resulting in less influence of environment. It is noted that when the ratio of KH550/HTPMS was 0.3:0.7, the release percent of nitrogen at the first 15 days were the slowest constant and the release percent reached 50 percent after 30 days. As the water permeability of the coating material decreased, the hindrance of water entering the urea particles through the polyurethane coating increased, hence the longer of urea release period. The coated fertilizer has great potential research implication compared to some current studies where hydroxyl-terminated dimethyl silicone modified transgenic soybean oil as an bio-based coating materials for controlled-release urea fertilizers, in which the nitrogen release reached 60 percent after 30 days [31], graphene oxide/polymer latex composite films coated on KNO_3_ fertilizers, in which the fertilizer release reached 70 percent after 30 days [12] and siloxane/polyether dual modified biopolymers coated fertilizers, in which the fertilizers release released 75 percent after 30 days [34].

### 3.8. Surface Microscopic Morphology of Coated Materials before and after PCUs Release

**Figure 12:**
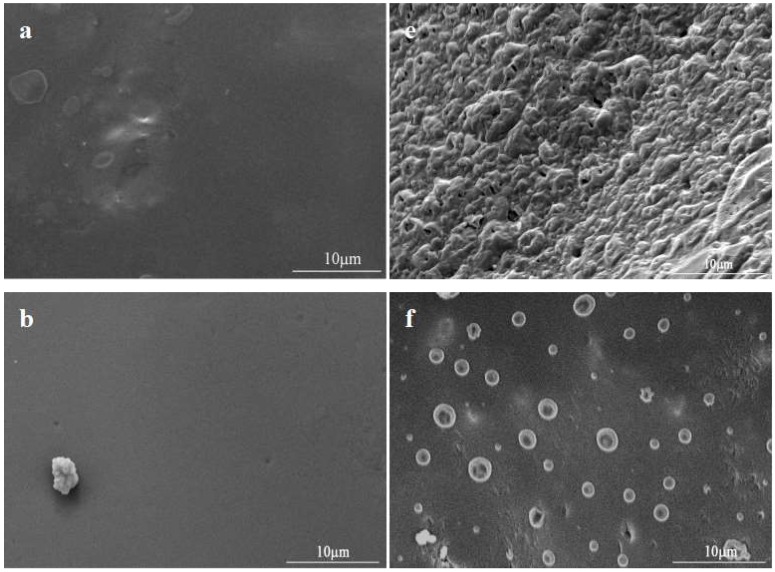

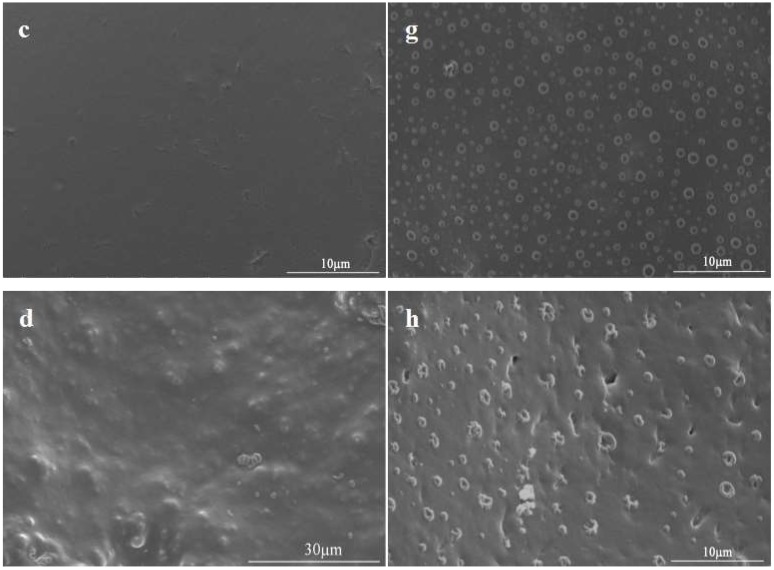
The surface SEM images of the coated materials before and after PCU release in aqueous solution. Before release: (**a**) 0, (**b**) 0.1:0.9, (**c**) 0.3:0.7, and (**d**) 0.5:0.5; After release: (**e**) 0, (**f**) 0.1:0.9, (**g**) 0.3:0.7, and (**h**) 0.5:0.5. (The data ratio is the KH550 to HTPMS content ratio.)

SEM images (Figure 12) illustrated the surface of the unmodified and KH550/HTPMS modified PCU shells. The surface of PCU became much rougher (Figure 12b–d) after the silicon modification; meanwhile, the surface smoothness of the modified shell was slightly reduced with the increase of the content of KH550. The surface microscopic morphologies of the coating material after the release of PCUs in aqueous solution are shown in Figure 12e–h. Compared to the modified shells, the surface of the unmodified shells was rougher and with lumps, swelling significantly, and there were obvious holes after the release of urea which might be easier to allow water to enter the shell to dissolve the urea for N release. However, the surface roughness of the modified polyurethane coating decreases obviously after the urea release. 

In addition, the surface in Figure 12f consists some small pores with uniform size and distribution, so the urea release is greatly hindered and the release rate is slow in the process. In Figure 12g, the surface pores of the PCU shells are the smallest, the size and distribution are more uniform, and the hole is almost invisible; hence the urea is the biggest obstacle in the release process where the release of N in the urea and the release rate is the slowest. Compared to the Figure 12g, the surface pores of the PCU shells are larger and smaller in Figure 12h, and the holes are obvious, so that the hindrance in the urea release process is smaller. These results are consistent with the cumulative release curve of N. The holes of polyurethanes were obviously decreased after the modification of the silicon, which may be the three-dimensional networks formed, and the urea is hindered in different degrees during the release process, thus slowing down the release rate of urea. With the size and distribution being more uniform with the pores in the polyurethane surface, the coating material had a lower water permeability and the water entering the particles through the coating was even more obstructed, so it took longer to dissolve the urea core. Therefore, the release period of N was longer. 

## 4. Conclusions

In summary, a novel bio-based PUs was fabricated from recycled oil modified by double organic silicone and utilized as coating films for controlled-release fertilizers. The slow and constant nitrogen release behaviors from the fertilizer could be attributed to the lower water permeability, higher hydrophobicity, and higher thermal stability of the PU coatings which were modified by KH550/HTPMS. The optimal weight ratio for KH550/HTPMS was 0.3:0.7 because of the slow release profile. The overall results showed that the recycled oil could be used in the slow release fertilizer, which not only reduced the loss of nutrients and prolonged the fertilizer cycle but also reduced the waste and improved the agricultural ecological environment.

## Figures and Tables

**Table 1:** The elemental compositions of PUs before and after the modification by double organic silicone.

Sample	Chemical Composition/%				Atom Ratio/%		
	C	O	N	Si	O/C	N/C	Si/C
PUs	82.56	14.68	2.76	-	17.78	3.34	-
K-H-PUs	69.64	16.92	3.93	9.51	24.30	5.64	13.66

**Table 2:** The C1s fitting results of the surface before and after modification of the PUs.

PUs			K-H-PUs		
Linkage	Binding Energy (eV)	Atomic %	Linkage	Binding Energy (eV)	Atomic %
C–C	284.80	43.70	C–C	284.80	44.62
C–N	286.17	14.80	C–N	286.15	16.05
C–O	284.20	28.97	C–O	284.21	24.55
–COO	288.52	12.53	–COO	289.13	14.78

**Table 3:** The influence of the mass ratio of KH550/HTPMS on the thermal stability of PCU.

KH550:HTMS				
Content (%)	*T*_5%_ (°C)	*T*_50%_ (°C)	*T*_max1_ (°C)	*T*_max2_ (°C)
0.1:0.9	188.4	243.4	244.7	356.7
0.2:0.8	195.7	258.7	248.7	358.7
0.3:0.7	190.6	284.6	241.6	359.6
0.4:0.6	190.7	246.7	242.4	371.4
0.5:0.5	192.6	327.6	238.6	354.5
